# Platelet Expression of Stromal Cell-Derived Factor-1 Is Associated with the Degree of Valvular Aortic Stenosis

**DOI:** 10.1371/journal.pone.0097405

**Published:** 2014-05-16

**Authors:** Thomas Wurster, Roland Tegtmeyer, Oliver Borst, Dominik Rath, Tobias Geisler, Meinrad Gawaz, Boris Bigalke

**Affiliations:** 1 Medizinische Klinik III, Kardiologie und Kreislauferkrankungen, Eberhard-Karls-Universität Tübingen, Germany; 2 King's College London, Division of Imaging Sciences and Biomedical Engineering, London, United Kingdom; University Hospital Medical Centre, Germany

## Abstract

**Background and Purpose:**

Platelet surface expression of stromal-cell-derived factor-1 (SDF-1) is increased during platelet activation and constitutes an important factor in hematopoetic progenitor cell trafficking at sites of vascular injury and ischemia. Enhanced platelet SDF-1 expression has been reported previously in patients suffering from acute coronary syndrome (ACS). We hypothesized that expression of platelet associated SDF-1 may also be influenced by calcified valvular aortic stenosis (AS).

**Methods:**

We consecutively evaluated 941 patients, who were admitted to the emergency department with dyspnea and chest pain. Platelet surface expression of SDF-1 was determined by flow cytometry, AS was assessed using echocardiography and hemodynamic assessment by heart catheterization. A 1∶1 propensity score matching was implemented to match 218 cases with 109 pairs adjusting for age, sex, cardiovascular risk factors, and medication including ACE inhibitors, angiotensin receptor blockers, beta blockers, statins, aspirin, clopidogrel, GPIIb/IIIa antagonists, and vitamin K antagonists.

**Results:**

Patients with valvular AS showed enhanced platelet SDF-1 expression compared to patients without AS (non-valvular disease, NV) independent of ACS and stable coronary artery disease (SAP) [mean fluorescence intensity (MFI) for ACS (AS vs. NV): 75±40.4 vs. 39.5±23.3; P = 0.002; for SAP (AS vs. NV): 54.9±44.6 vs. 24.3±11.2; P = 0.008]. Moreover, the degree of AS significantly correlated with SDF-1 platelet surface expression (r = 0.462; P = 0.002).

**Conclusions:**

Valvular AS is associated with enhanced platelet-SDF-1 expression; moreover the degree of valvular AS correlates with SDF-1 platelet surface expression. These findings may have clinical implications in the future.

## Introduction

Degenerative calcified valvular heart disease concerns a noteworthy group of patients in the Western world and increases with age. The more frequent appearance of aortic stenosis (AS) in an increasingly elderly population poses a growing challenge to clinicians and public healthcare [1]. Risk factors for the development of AS are similar to those associated with atherosclerosis, and approximately half of the patients with severe AS feature significant coronary artery disease (CAD) [2]. Nevertheless, patients with aortic sclerosis are also likely to suffer from cardiovascular events [3]. To date, biomarkers play a subordinate role in the diagnosis and staging of AS. The chemokine stromal cell-derived factor-1 (SDF-1) captures an important role in the regeneration of ischemic tissue [4] and stem cell trafficking [5]. Both, in patients with AS [6] and acute coronary syndrome (ACS) [7] platelets show increased reactivity. However, platelets exhibit an enhanced SDF-1 surface expression upon activation [8], [9]. In a previous study, in a large cohort comprising 1,000 patients suffering from acute chest pain, our group demonstrated an enhanced SDF-1 expression on activated platelets in patients with ACS [10]. Hemodynamic alterations caused by AS are likely to cause platelet activation, therefore platelet-SDF-1 surface expression might be associated with AS. The aim of the present study was to evaluate platelet SDF-1 surface expression in patients presenting symptomatic CAD and concomitant AS in the emergency care unit.

## Methods

### Study population and enrolment criteria

We consecutively evaluated a cohort of 941 patients, admitted for chest pain and/or dyspnea to the emergency care unit at the University Hospital Tübingen, all of whom underwent coronary angiography and complete hemodynamic assessment by heart catheterization and echocardiographic analysis. After implementation of a 1∶1 propensity score matching adjusting for age, sex, cardiovascular risk factors and medication including ACE inhibitors, angiotensin receptor blockers, beta blockers, statins, aspirin, clopidogrel, GPIIb/IIIa antagonists, and vitamin K antagonists, 218 cases with 109 pairs were matched. All patients underwent ECG and serum examination for troponin-I, creatine kinase, C-reactive peptide and creatinine, measurement of blood pressure, clinical examination and echocardiography, as well as left heart catheterization. Exclusion and inclusion criteria are given in *Table 1*. All participant patients provided written informed consent. The study was conducted according to the principles of the Declaration of Helsinki and approved by the local ethics committee at the University Hospital Tübingen, Germany (Ethics approval number: “292/2005V platelet activation in patients with coronary artery disease“).

**Table 1:** Inclusion and Exclusion Criteria.

Inclusion criteria	Patients with emergency care unit admittance due to chest pain and/or dyspnea and further assessment including coronary angiography, complete hemodynamic assessment by heart catheterization and echocardiographic analysis
Exclusion criteria	Additional hemodynamically significant valve lesions (moderate or severe) or patients with (paradoxical) low flow/low gradient aortic stenosis, age below 18 years, cardiac shock, stroke, malignancy, connective tissue disease, psychiatric disease, anorexia, pregnancy or hormone replacement therapy in women, lack or inability of informed consent

### Definitions

ACS: ACS definition according to AHA/ACC guidelines [11]: ACS comprises ST-Elevation Myocardial Infarction (STEMI), non–ST-segment elevation myocardial infarction (NSTEMI) as well as unstable angina pectoris. The predetermined diagnostic threshold for troponin-I was ≥0.04 µg/L [12].

SAP: Patients suffering from stable angina pectoris manifesting through chest pain with stable intensity and stress inducible myocardial ischemia [13]. Furthermore patients showing CAD and presenting with non-elevated CK or troponin-I and without dynamic ECG changes.

AS: AS definition according to AHA/ACC guidelines: Mild AS (AS I°) exhibits an aortic valve area (AVA) of more than 1.5 cm^2^ with a mean gradient of less than 25 mmHg, or a jet velocity less than 3.0 m per second. Moderate AS (AS II°) exhibits an AVA of 1.0 to 1.5 cm^2^ with a mean gradient of 25 to 40 mmHg, or a jet velocity of 3.0 to 4.0 m per second. Severe AS (AS III°) exhibits an AVA of less than 1.0 cm^2^ with a mean gradient greater than 40 mmHg, or a jet velocity greater than 4.0 m per second [14].

### Sample collection, FACS analysis and AVA determination

On admission to the emergency care unit venous blood samples were drawn from patients with chest pain and dyspnea while standard routine laboratory assessment and filled into 5 mL citrate phosphate dextrose adenine vials (CPDA). Blood samples have been processed within 3 hours maximum after blood was drawn from the participant according to a predefined assay as described before [15], [16]. In brief, CPDA-Blood was diluted with phosphate buffered saline (PBS) in relation 1∶50. From this suspension, 35 µl were put into test tubes and further 5 µl PBS were added. Then 5 µl solute containing either FITC labeled anti-SDF-1 or anti-CD62P antibodies following 5 µl of PE-labeled anti-CD42b antibodies were adjusted. The probes were incubated for 25 minutes at 21°Celcius in complete darkness. Afterwards 300 µl of 0.5% paraformaldehyde were added. The probes were stored at 4°Celcius in complete darkness not later than 48 hours until two-color whole blood flow cytometry analysis was performed (FACS-Calibur Flow Cytometer, Becton-Dickinson, Heidelberg, Germany). Platelet surface expression of SDF-1, CD62P and GPIb (CD42) was analyzed using conjugated antibodies [SDF-1, R&D Systems, Minneapolis, MN, USA; clone 79014; fluorescein isothiocyanate (FITC); CD62P, Immunotec, Marseille, France; clone CLB-Thromb/6; FITC and CD42b, Immunotec, Marseille, France; clone SZ2; phycoerythrin (PE)]. CD42b-PE was used for identification of the platelet population as second color control antibody. The mean fluorescence intensity (MFI) served as quantity index of biomarker surface expression. AVA was estimated using echocardiography (iE33, Philips Medical Systems) according to the continuity equation including the velocity-time integral of the aortic and left ventricular outflow tract flows.

### Statistics

We considered a probability value of less than 0.05 as statistically significant. Propensity score was applied for bias reduction. Therefore, we implemented a 1∶1 propensity score matching to balance 109 cases with 109 controls regarding age, sex, cardiovascular risk factors and medication including ACE inhibitors, angiotensin receptor blockers, beta blockers, statins, aspirin, clopidogrel, GPIIb/IIIa antagonists and vitamin K antagonists. Raynald's SPSS tool for propensity score matching was used. We analyzed Categorical data by chi-square test. For comparison of differences between groups Kruskal–Wallis test was used for non-normally distributed data. For evaluation of associations concerning events and variables a log-rank test (Mantel-Cox) was implemented. Correlation between AS and SDF-1 or CD62P expression was analyzed using Pearson correlation. All statistical analyses were performed using SPSS version 19.

## Results

**Figure 1:**
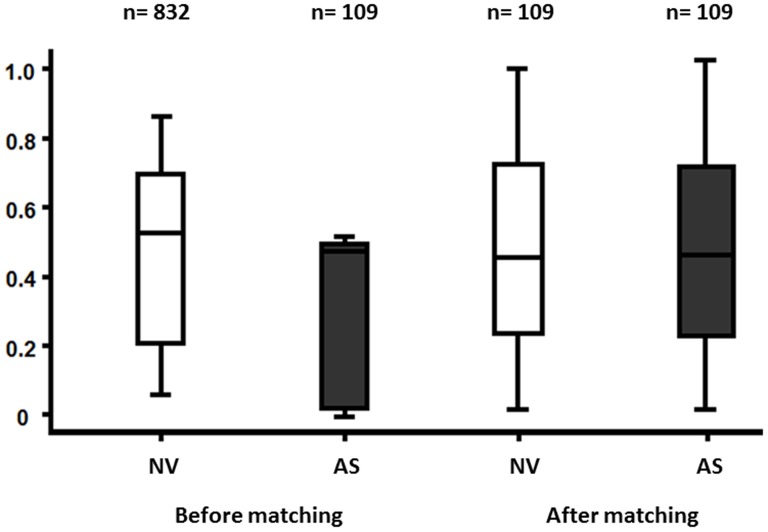
Propensity score matching of patients with aortic stenosis (AS) and non-valvular (NV) disease. Implementation of a 1∶1 propensity score matching. 218 cases with 109 pairs comprising age, sex, cardiovascular risk factors and medication regarding ACE inhibitors, angiotensin receptor blockers, beta blockers, statins, aspirin, clopidogrel, GPIIb/IIIa antagonists and vitamin K antagonists were matched.

A consecutive cohort of 941 patients with underlying coronary artery disease [ACS,n = 498; SAP,n = 443] suffering from chest pain and dyspnea was evaluated on hospital admission. All patients were evaluated by heart catheterization and transthoracic echocardiography. Platelet surface expression of SDF-1 was determined by flow cytometry. Finally, a 1∶1 propensity score matching was implemented to match 218 cases with 109 pairs comprising age, gender, cardiovascular risk factors, as well as medication including ACE inhibitors, angiotensin receptor blockers, beta blockers, statins, aspirin, clopidogrel, GPIIb/IIIa antagonists and vitamin K antagonists (*Fig. 1*). The demographic details are given in *Table 2*.

**Table 2:** Baseline Patients' Characteristics and Medical Treatment on Admission.

Characteristics	Before Matching	After Matching	
	Total (n = 941)	Non-valvular Disease (n = 109)	Aortic Stenosis (n = 109)
Age – years	65.5	66.7	62.7
Gender – no. (%)			
Female	636 (67.6%)	79 (72.5%)	76 (69.7%)
Male	305 (32.4%)	30 (27.5%)	33 (30.3%)
ACS	613 (65.1%)	87 (79.8%)	55 (50.5%)
SAP	328 (34.9%)	22 (20.2%)	54 (49.5%)
Cardiovascular Risk Factors – no. (%)			
Arterial hypertension	714 (75.9%)	81 (74.3%)	78 (71.6%)
Hyperlipidaemia	506 (53.8%)	59 (54.1%)	62 (56.9%)
Diabetes	253 (26.9%)	22 (20.2%)	36 (33%)
Family history of CVD	158 (16.8%)	11 (10.1%)	19 (17.4%)
Smoking	321 (34.1%)	47 (43.1%)	43 (39.4%)
Atrial fibrillation	147 (15.6%)	9 (8.2%)	22 (20.2%)
Medication – no. (%)			
ACE inhibitors	88 (9.4%)	22 (20.2%)	10 (9.2%)
Angiotensin receptor blockers	64 (6.8%)	7 (6.4%)	6 (5.5%)
Beta blockers	67 (7.1%)	7 (6.4%)	6 (5.5%)
Statins	67 (7.1%)	7 (6.4%)	6 (5.5%)
Aspirin	70 (7.4%)	7 (6.4%)	8 (7.3%)
Clopidogrel	66 (7%)	6 (5.5%)	7 (6.4%)
Vitamin K antagonists	67 (7.1%)	4 (3.7%)	5 (4.6%)
Medication on hospital discharge– no. (%)			
ACE inhibitors	682 (72.5%)	78 (71.6%)	80 (73.3%)
Angiotensin receptor blockers	172 (18.3%)	7 (6.4%)	8 (7.3%)
Beta blockers	777 (82.6%)	84 (77.1%)	79 (72.5%)
Statins	695 (73.9%)	86 (78.9%)	75 (68.8%)
Aspirin	762 (81%)	85 (78%)	77 (70.6%)
Clopidogrel	680 (72.3%)	82 (75.2%)	72 (66.1%)
Vitamin K antagonists	185 (19.7%)	3 (2.8%)	18 (16.5%)

**Figure 2:**
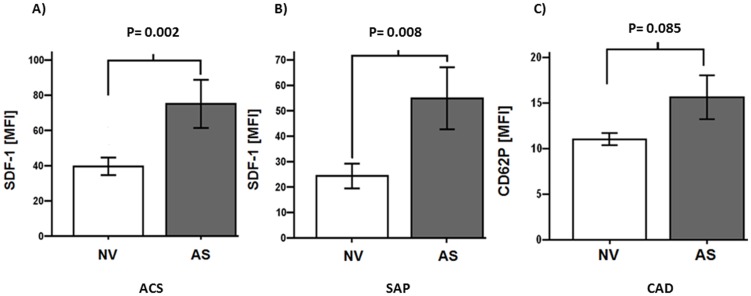
Platelet surface expression of SDF-1 in acute coronary syndrome (ACS) (A), stable angina pectoris (SAP) (B) subgroups and platelet CD62P expression in patients with symptomatic coronary artery disease (CAD) (C). (A, B) SDF-1 mean fluorescence intensity (MFI) in patients with non-valvular disease (NV) compared to patients with aortic stenosis (AS) [ACS (AS vs. NV): 75±40.4 vs. 39.5±23.3; P = 0.002; SAP (AS vs. NV): 54.9±44.6 vs. 24.3±11.2; P = 0.008]. (C) CD62P mean fluorescence intensity showed a trend for increased platelet activation in CAD patients with aortic stenosis (AS) compared to patients with non-valvular disease (NV) [AS vs. NV: 15.6±12.6 vs. 11±3.6; P = 0.085].

**Figure 3:**
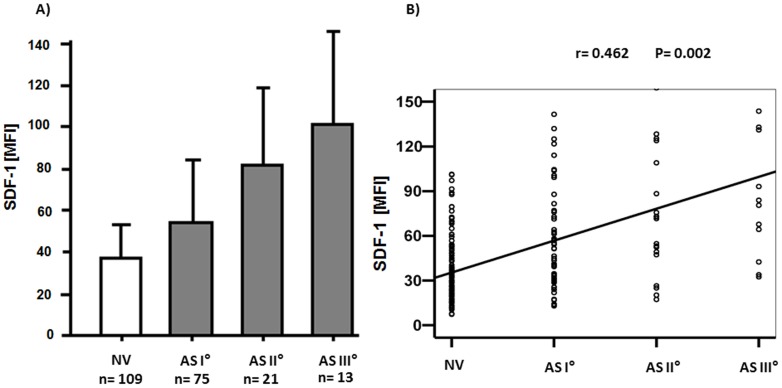
Platelet SDF-1 surface expression according to the degree of aortic stenosis (AS) (A) and association between platelet SDF-1 expression and the degree of AS (B). (A) Platelet SDF-1 surface expression depicted by mean fluorescence intensity (MFI± standard deviation) in patients with non-valvular disease (NV; n = 109; 36.3±22.1) and patients with mild (AS I°; n = 75; 54.2±42.2), moderate (AS II°; n = 21; 81.8±47.9) and severe (AS III°; n = 13; 100.7±55.9) aortic stenosis. (B) Association of platelet SDF-1 surface expression in patients with non-valvular disease (NV) and patients with mild AS (I°), moderate AS (II°) and severe AS (III°) (r = 0.462; P = 0.002).

Patients with valvular AS showed enhanced platelet SDF-1 expression compared to patients with non-valvular disease (NV) both with ACS and SAP [mean fluorescence intensity (MFI) for ACS (AS vs. NV): 75±40.4 vs. 39.5±23.3; P = 0.002; for SAP (AS vs. NV): 54.9±44.6 vs. 24.3±11.2; P = 0.008] (*Fig. 2A,B*). CD62P mean fluorescence intensity showed a trend for increased platelet activation in CAD patients with AS compared to non-valvular disease (NV) [AS vs. NV: 15.6±12.6 vs. 11±3.6; P = 0.085] (*Fig. 2C*). Among the 109 patients featuring AS, 75 were classified as AS I° (69%), 21 were classified as AS II° (19%) and 13 were classified as AS III° (12%) (*Fig. 3A*). The degree of AS correlated with SDF-1 platelet surface expression (r = 0.462; P = 0.002) (*Fig. 3B*).

## Discussion

The major findings of the present study are: 1) Patients with valvular AS show enhanced platelet SDF-1 expression compared to patients with non-valvular disease both in patients with ACS and SAP; 2) The degree of AS correlates with surface expression of platelet SDF-1.

SDF-1, also known as CXCL12 is a prominent CXC-chemokine that binds to its receptors CXCR4 and CXCR7 and regulates chemotaxis, migration and differentiation of inflammatory cells including monocytes and hematopoetic progenitor cells [9]. Among other cells, platelets have been shown to store and release substantial amounts of SDF-1 upon activation [9], [17], [18]. In resting platelets, SDF-1 is stored in cytosolic granules. Upon platelet activation and peripheralization of granules, a markedly enhanced platelet surface expression of SDF-1 can be observed [8]. Previous studies demonstrated a positive correlation between plasma levels of SDF-1 and platelet SDF-1 surface expression [19], [20]. However, experimental data suggest an imprecise relation between SDF-1 detachment and SDF-1 expression [8]. Therefore, the study focused on platelet associated SDF-1 expression.

In a large study including patients suffering from chest pain, our group demonstrated an enhanced SDF-1 expression on the platelet surface in patients with ACS [10]. Moreover, our group observed an association between enhanced platelet SDF-1 expression with reduced left ventricular function and the number of circulating CD34+ progenitor cells, predominantly in patients suffering from acute myocardial infarction (MI) [19], [21].

Circulating platelets are critically influenced by altered hemodynamics and increased shear stress in individuals with AS [6], [22]–[25], moreover high shear forces *in vivo* and *in vitro* are associated with platelet activation [26]. Experimental data suggests the release of antithrombotic agents, such as nitric oxide (NO) and prostacyclin from normal aortic valves [27], [28], whereas increased platelet reactivity as well as thrombus formation have been observed on severely calcified and stenotic aortic valves [29]. In previous studies the expression of several biomarkers in patients suffering from AS has been observed. Dimitrow et al. showed enhanced concentrations of thrombin, thrombin–antithrombin complexes (TAT), prothrombin fragment 1+2 (F1+2), soluble CD40 ligand (sCD40L) and beta-thromboglobulin (beta-TG) in patients with AS [30]. Furthermore, Luszczak et al. observed detectable plasma tissue factor (TF) and factor XIa activity associated with thrombin generation in patients with especially severe AS [31]. Increased plasma thrombin formation and platelet activation in patients with moderate to severe AS has also been reported by Natorska et al. in patients additionally deficient for high molecular weight multimers of von Willebrand factor (HMWM vWF) [24]. In fact, platelet activation via thrombin receptor PAR-1, as well as adenosine diphosphate (ADP) receptors P2Y1/P2Y12 and glycoprotein VI (GPVI)-dependent pathways result in increased platelet surface expression and release of SDF-1 [8]. Compared to our preceding study in patients with ACS, subgroup analysis in the present study reveals an even more increased platelet SDF-1 expression in patients with ACS featuring AS compared to patients with ACS and non-valvular disease. Coherently, platelet SDF-1 expression is enhanced in patients with SAP and AS compared to non-valvular SAP. Therefore, AS resembles an independent co-variate associated with systemic platelet activation. The present study revealed moreover a significant correlation between increased SDF-1 platelet surface expression and the degree of AS. Thus, high shear stress associated with AS may be a determining factor regarding platelet activation. Previous studies revealed an association between high shear stress featuring AS and platelet aggregation due to increased binding of plasma HMWM of vWF to the platelet membrane [23], [32]. Comparatively elevated shear stress due to obstruction of the left ventricular outflow tract in hypertrophic obstructive cardiomyopathy is also associated with platelet activation and augmented thrombin generation [33].

In the present study, in contrast to SDF-1, platelet expression of CD62P in CAD patients showed merely a trend for increased platelet surface expression in patients with AS compared to NV (P = 0.085). Correspondingly, previous studies also revealed only weak associations between CD62P and AS severity or elevated gradients of the left ventricular outflow tract [24], [33]. Despite evaluation of several biomarkers in AS, currently no biomarker for AS detection, progression or prognosis is used in clinical routine so far. However, there are several promising biomarkers which might provide future diagnostic and therapeutic implications [34]. To our knowledge, this is the first study demonstrating a significant association between platelet SDF-1 expression and the severity of AS in symptomatic patients. Thus, biomarker panels including platelet associated SDF-1 may offer new diagnostic options in diagnosis and staging of patients with AS in the future and might be especially interesting in the emergency setting. Further studies as well as simplified assays are needed to assess the impact of platelet SDF-1 expression in patients with AS especially in the preclinical setting.

## Conclusions

To date, clinical examination, echocardiography and heart catheterization represent common standard in AS detection and staging. Our study shows increased platelet SDF-1 expression in patients with AS compared to patients with non-valvular disease both in ACS and SAP in a large cohort of patients after admittance to the emergency care unit. Therefore, detection of enhanced platelet SDF-1 expression in patients with chest pain or dyspnea on hospital admission might raise the suspicion of AS as well as concomitant ACS. In conclusion, platelet SDF-1 might represent a novel biomarker in AS to indicate risk and therefore should be evaluated for its prognostic value in aortic valve disease.
